# Utility of the inspiratory phase in high-resolution computed tomography evaluations of pediatric patients with bronchiolitis obliterans after allogeneic bone marrow transplant: reducing patient radiation exposure

**DOI:** 10.1590/0100-3984.2015.0181

**Published:** 2017

**Authors:** Paulo Henrique Togni Filho, João Luiz Marin Casagrande, Henrique Manoel Lederman

**Affiliations:** 1 MD, MSc, Attending Physician, Department of Diagnostic Imaging, Escola Paulista de Medicina da Universidade Federal de São Paulo (EPM-Unifesp), São Paulo, SP, Brazil; 2 Radiologist, Fellow in Musculoskeletal Imaging, Instituto de Radiologia do Hospital das Clínicas da Faculdade de Medicina da Universidade de São Paulo (InRad/HC-FMUSP), São Paulo, SP, Brazil; 3 Tenured Full Professor, Department of Diagnostic Imaging, Escola Paulista de Medicina da Universidade Federal de São Paulo (EPM-Unifesp), São Paulo, SP, Brazil

**Keywords:** Bronchiolitis obliterans, Radiation dosage, Bone marrow transplantation, Tomography, X-ray computed

## Abstract

**Objective:**

To evaluate the utility of the inspiratory phase in high-resolution computed tomography (HRCT) of the chest for the diagnosis of post-bone marrow transplantation bronchiolitis obliterans.

**Materials and Methods:**

This was a retrospective, observational, cross-sectional study. We selected patients of either gender who underwent bone marrow transplantation and chest HRCT between March 1, 2002 and December 12, 2014. Ages ranged from 3 months to 20.7 years. We included all examinations in which the HRCT was performed appropriately. The examinations were read by two radiologists, one with extensive experience in pediatric radiology and another in the third year of residency, who determined the presence or absence of the following imaging features: air trapping, bronchiectasis, alveolar opacities, nodules, and atelectasis.

**Results:**

A total of 222 examinations were evaluated (mean, 5.4 ± 4.5 examinations per patient). The expiratory phase findings were comparable to those obtained in the inspiratory phase, except in one patient, in whom a small uncharacteristic nodule was identified only in the inspiratory phase. Air trapping was identified in a larger number of scans in the expiratory phase than in the inspiratory phase, as was atelectasis, although the difference was statistically significant only for air trapping.

**Conclusion:**

In children being evaluated for post-bone marrow transplantation bronchiolitis obliterans, the inspiratory phase can be excluded from the chest HRCT protocol, thus reducing by half the radiation exposure in this population.

## Resumo

**Objetivo:**

Avaliar a necessidade da fase inspiratória na tomografia computadorizada de alta resolução (TCAR) do tórax no diagnóstico de bronquiolite obliterante pós-transplante de medula óssea.

**Materiais e Métodos:**

Estudo retrospectivo, observacional, transversal. Foram selecionados pacientes que realizaram transplante de medula óssea e TCAR do tórax, com idades entre 3 meses e 20,7 anos, de ambos os sexos, durante 12 anos e 9 meses (de 1º de março de 2002 a 12 de dezembro de 2014). Todos os exames foram realizados com qualidade técnica adequada para análise pelos radiologistas. As imagens foram analisadas em consenso por um radiologista com grande experiência em radiologia pediátrica e um radiologista em treinamento, avaliando aspectos específicos das imagens como aprisionamento aéreo, bronquiectasia, opacidade, nódulos inespecíficos e atelectasia, com critérios objetivos.

**Resultados:**

Foram avaliados 222 exames (média de 5,4 ± 4,5 exames por paciente). A fase expiratória demonstrou os mesmos achados que as duas fases em conjunto, exceto por um único nódulo identificado somente na fase inspiratória. A fase expiratória identificou um número estatisticamente superior de aprisionamento aéreo em relação à fase inspiratória e um número maior de atelectasia, porém sem diferença significativa.

**Conclusão:**

A fase inspiratória pode ser excluída do protocolo para avaliação de crianças pós-transplante de medula óssea com suspeita de bronquiolite obliterante, reduzindo, assim, pela metade a quantidade de radiação à qual essas crianças são expostas.

## INTRODUCTION

Bronchiolitis obliterans (BO) is a generic term used in order to describe the inflammation of the small airways, defined as those with a diameter of less than 2 mm and with no cartilage in their walls^(1)^. It is an obstructive airway disease, caused by a wide variety of conditions, such as connective-tissue diseases, inhalation of toxins, infections, and drug use^(2)^. BO is associated with high mortality rates, ranging from 21% to 100%^(3-9)^.

BO is the most common noninfectious late pulmonary complication of allogeneic bone marrow transplantation (ABMT) and the one with the worst prognosis, usually occurring more than 100 days after transplantation^(10-12)^. In the first study of post-ABMT BO, conducted in 1982^(13)^, lymphocytic bronchiolitis was found in 10% of the autopsies of patients who died after ABMT.

The clinical course of BO includes irreversible and progressive airway obstruction, and the treatment is aimed at stabilizing the forced expiratory volume in one second (FEV1). According to The International Bone Marrow Transplantation Registry, the incidence of BO is 1.7% in the first two years after ABMT, BO having been identified in 6275 patients who underwent ABMT with a compatible donor^(9)^, and the disease is rare among patients who undergo autologous transplantation^(14-16)^.

The symptoms of BO are often insidious at their onset and usually include cough (60–100%), dyspnea (50–70%), wheezing and reduced breath sounds^(4,6,17,18)^. Pulmonary function tests show reduced FEV1 and FEV1/forced vital capacity ratio.

The risk factors associated with post-ABMT BO are shown in Table 1^(3-8,17,19-28)^. The most important associated risk factor is the presence of chronic graft-versus-host disease (GVHD)^(4,21)^.

**Table 1:** Risk factors associated with post-ABMT BO.

Feature	Risk factor
Consistent	Allogeneic hematopoietic stem cell transplantation
	Chronic and progressive graft-versus-host disease
Likely	*De novo *or quiescent-type chronic graft-versus-host disease
	Donor with advanced age
	Airflow obstruction before ABMT
	History of viral airway infection
Possible	Acute graft-versus-host disease
	Busulfan-based regimens
	Full-body irradiation
	Methotrexate-based graft-versus-host disease prophylaxis
	Hypogammaglobulinemia
	Cytomegalovirus infection
	Donor with advanced age
	Associated diseases (e.g., chronic myeloid leukemia)
	Gastroesophageal reflux disease

ABMT, allogeneic bone marrow transplantation.

Chien et al.^(28)^ found an attributable mortality of 9% in 3 years, 12% in 5 years, and 18% in 10 years after ABMT in patients with airflow obstruction, and it was statistically higher in patients with chronic GVHD (22% in 3 years, 27% in 5 years, and 40% in 10 years).

Given the severity of the disease and the fact that its presence increases the long-term mortality rates between those who undergo ABMT, more studies are needed to better define the clinical features of BO^(28)^.

The definitive diagnosis of BO is made by biopsy and histopathological examination^(11)^. However, high-resolution computed tomography (HRCT) of the chest plays an important role in diagnosing bronchiolar diseases, because they present nonspecific symptoms which usually appear only when advanced destruction of the peripheric airways has already become established^(1)^. Although the tomographic patterns are nonspecific, they are useful in showing which parts of the lungs are affected^(1)^, and they may also show associated conditions such as coexisting infections, BO organizing pneumonia, and idiopathic pneumonia syndrome^(26)^, thus narrowing down the differential diagnosis.

The current chest HRCT protocol for BO evaluation in pediatric patients is the same at that used for adults, including an inspiratory and an expiratory phase. In pediatric patients, the concerns about the use of ionizing radiation are even greater, particularly in post-ABMT patients, because they need follow-up CTs from early ages, which increases the risks of radiation-induced cancer^(10,29)^.

Given these concerns in reducing the radiation exposure in these children and the fact that one of the most important imaging features in BO is air trapping secondary to the airway obstruction, which is best seen in the expiratory phase, the real need for a chest HRCT protocol including the inspiratory phase when evaluating these patients has yet to be proven.

Our aim was to evaluate the usefulness of the chest HRCT inspiratory phase for the diagnosis of BO in post-ABMT patients, considering additional findings that would not be detected in the expiratory phase and the implications for clinical decision-making.

## MATERIALS AND METHODS

This was a retrospective, observational, cross-sectional study conducted in the Diagnostic Imaging Department of the Escola Paulista de Medicina da Universidade Federal de São Paulo and at the Instituto de Oncologia Pediátrica/Grupo de Apoio a Criança com Câncer (IOP/GRAACC, Pediatric Oncology Institute/Support Group for Children with Cancer) and was approved by the research ethics committee of the institution.

We selected consecutive patients who underwent ABMT and HRCT of both genders, between March 1, 2002 and December 12, 2014. Patient ages ranged from 3 months to 20.7 years. The diagnosis of BO was based on clinical and biochemical data, as well as on the results of functional tests and on patient medical history. All patients were diagnosed at least 90 days after ABMT, the mean time from ABMT to diagnosis being 180 days. Other complications were excluded on the basis of the natural history of the disease and physical examination. None of the patients underwent CT before ABMT, because CT of the chest is not indicated in asymptomatic patients with normal chest X-rays. None of our patients had reported pulmonary disease. All examinations were performed at the IOP/GRAACC Diagnostic Imaging Center, which is a referral center for pediatric cancer. We included only the studies in which the imaging technique was considered appropriate for reading.

**Figure 1:**
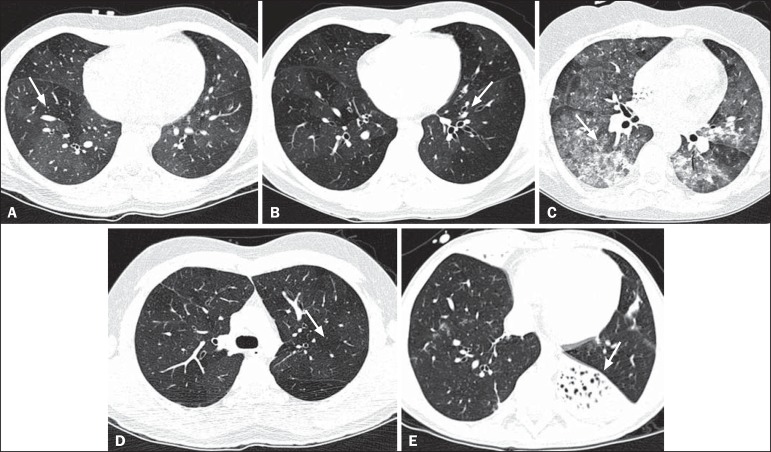
Chest HRCT, expiratory phase, showing air trapping (arrow in **A**), bronchiectasis (arrow in **B**), alveolar opacities (arrow in **C**), nonspecific nodules (arrow in **D**), and atelectasis (arrow in **E**).

The images were acquired on a dual-slice CT scanner (MX8000 Dual; Philips, Best, The Netherlands) with volumetric acquisition, a slice thickness of 1 mm, and an interslice gap of 8 mm. In most of the cases, the voltage and current were set to 120 kV and 130 mAs, respectively, yielding the same dose of radiation (2.4 mSv) in the inspiratory and expiratory phases, regardless of whether the acquisition was dynamic or (in older children) static. The images were reviewed by two radiologists: one was a radiologist with extensive experience in pediatric radiology; and the other was a third-year radiology resident. Initially, the radiologists read the images acquired in both phases (inspiratory and expiratory), seeking to identify the presence or absence (all qualitative measurements) of air trapping (Figure 1A), bronchiectasis (Figure 1B), alveolar opacities (Figure 1C), nodules (Figure 1D), and atelectasis (Figure 1E).

We defined air trapping as differing degrees of attenuation within the lung parenchyma-decreased attenuation (areas that are darker than the rest of the parenchyma) indicating the areas of air trapping. The thickening of the bronchial walls was assessed subjectively. In the cases of bronchiectasis, the radiologists applied general criteria such as bronchial diameter ≥ 1.5× that of the adjacent pulmonary artery, bronchial diameter ≥ 2.0 cm, and image of the bronchus approaching the peripheral lung parenchyma (< 1.0 cm from the adjacent pleural or mediastinal pleura). At another time point, the expiratory phase was analyzed separately, in order to identify those same imaging features. It was understood that if there were disagreements between the two radiologists, the opinion of the most experienced radiologist would prevail. However, there was no such disagreement.

In children who were uncooperative (those under six years of age), the inspiratory and expiratory phases were obtained bilaterally in the lateral decubitus position, the side in contact with the litter corresponding to the expiratory phase and the other side corresponding to the inspiratory phase.

Data were analyzed by descriptive statistics, expressed as absolute and relative frequencies, as well as by inferential statistics, with either the chi-square test or Fisher's exact test, together with the Z-test for comparisons between two sample proportions.

The statistical analysis was performed with the Statistical Package for the Social Sciences, version 16.0 (SPSS Inc., Chicago, IL, USA), and values of *p* < 0.05 were considered statistically significant.

## RESULTS

During the study period, 55 patients underwent ABMT and chest HRCT for the evaluation of BO. Of those 55 patients, 15 (27.3%) were excluded because their examinations were technically poor (available only on paper or film) or had not been performed at the IOP/GRAACC. Therefore, the final sample comprised 40 patients were included, ranging in age from 3 months to 20.7 years (mean, 9.7 ± 5.4 years). Those 40 patients underwent a total of 222 chest HRCT scans (mean, 5.4 ± 4.5 scans per patient), all of which were reviewed.

**Figure 2:**
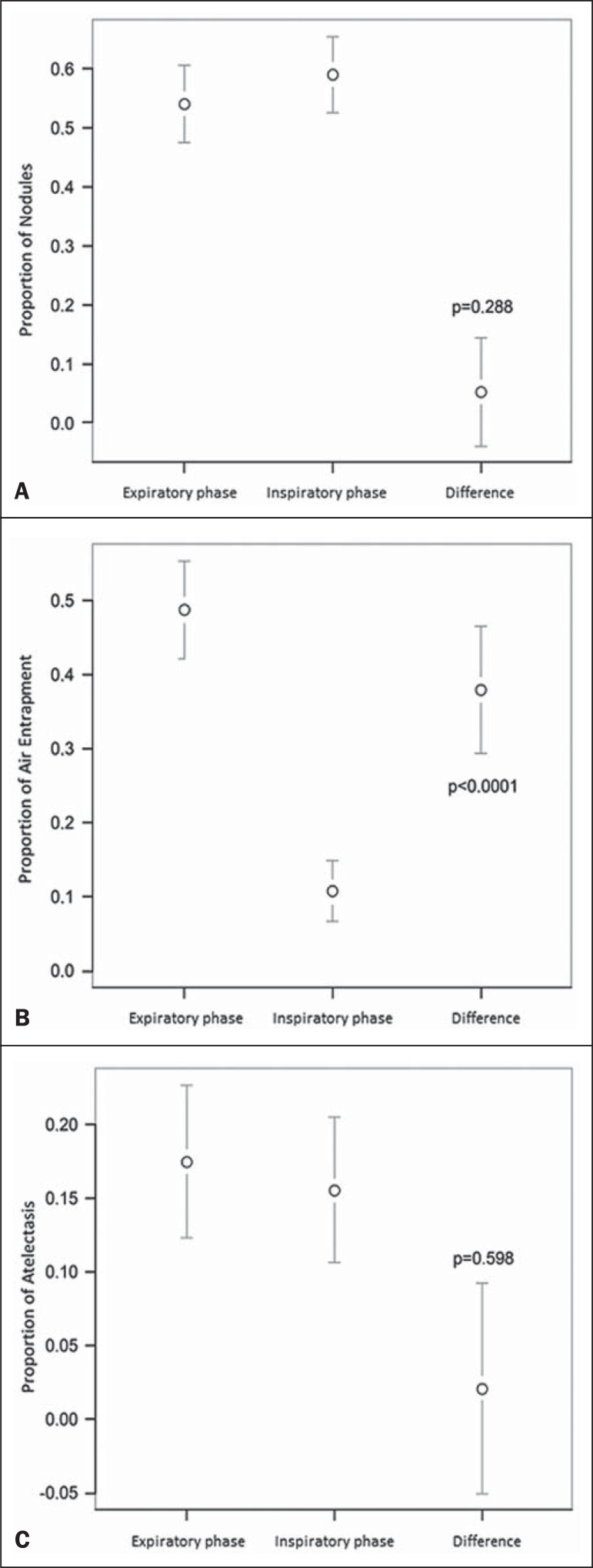
Graphics showing the difference between the inspiratory and expiratory phases in terms of the sample proportions for the findings of nodules (**A**), air trapping (**B**), and atelectasis (**C**).

Table 2 shows the main imaging findings after the inspiratory and expiratory phases had been analyzed (together and separately). The expiratory phase findings were the same as those obtained when the phases were analyzed together, except in one scan, in which a nonspecific nodule (of no clinical significance) was found only in the inspiratory phase (Figure 2A). However, there was no statistically difference between the findings as analyzed by the Z-test (*p* = 0.288). Nevertheless, the number of examinations in which air trapping was identified (Figure 2B) was higher for the expiratory phase than for the inspiratory phase, as was the number of examinations in which atelectasis was present (Figure 2C), although the difference was statistically significant only for the former comparison (*p* < 0.0001 and *p* = 0.598, respectively).

**Table 2:** Frequency of imaging findings when the inspiratory and expiratory phases were analyzed together and separately in 40 patients (*n* = 222 examinations).

	Phases							
	Inspiratory + Expiratory			Inspiratory			Expiratory	
Imaging feature	N	%		N	%		N	%
Air trapping	108	48.6		108	48.6		24	10.8
Bronchiectasis	107	48.2		107	48.2		107	48.2
Opacities	35	15.8		35	15.8		35	15.8
Nonspecific nodules	13	5.9		12	5.4		13	5.9
Atelectasis	36	16.2		36	16.2		32	14.4

Tables 3 to 7 show the statistically significant relationships between analyzing the phases together and analyzing them separately, in terms of the findings of air trapping, bronchiectasis, opacities, atelectasis, and nonspecific nodules, respectively.

**Table 3:** Comparison between the analysis of both phases together and that of the expiratory phase separately, in terms of the finding of air trapping, in 40 patients (*n* = 222 examinations).

Phase			
	Expiratory		
Inspiratory + Expiratory	Absent	Present	Total
Absent	114	0	114
Present	0	108	108
Total	114	108	222

**Table 4:** Comparison between the analysis of both phases together and that of the expiratory phase separately, in terms of the finding of bronchiectasis, in 40 patients (*n* = 222 examinations).

Phase			
	Expiratory		
Inspiratory + Expiratory	Absent	Present	Total
Absent	115	0	115
Present	0	107	107
Total	115	107	222

**Table 5:** Comparison between the analysis of both phases together and that of the expiratory phase separately, in terms of the finding of opacities, in 40 patients (*n* = 222 examinations).

Phase			
	Expiratory		
Inspiratory + Expiratory	Absent	Present	Total
Absent	187	0	187
Present	0	35	35
Total	187	35	222

**Table 6:** Comparison between the analysis of both phases together and that of the expiratory phase separately, in terms of the finding of atelectasis, in 40 patients (*n* = 222 examinations).

Phase			
	Expiratory		
Inspiratory + Expiratory	Absent	Present	Total
Absent	186	0	186
Present	0	36	36
Total	186	36	222

**Table 7:** Comparison between the analysis of both phases together and that of the expiratory phase separately, in terms of the finding of nonspecific nodules, in 40 patients (*n* = 222 examinations).

Phase			
	Expiratory		
Inspiratory + Expiratory	Absent	Present	Total
Absent	209	0	209
Present	1	12	13
Total	210	12	222

## DISCUSSION

Our study showed similar and statistically significant imaging findings when both phases were analyzed together and when only the expiratory phase was analyzed. Air trapping was detected significantly more often in the expiratory phase than in the inspiratory phase. When the phases were analyzed separately, atelectasis was detected more often in the expiratory phase and one nonspecific nodule was detected only in the inspiratory phase, although those differences were not statistically significant.

The aim of this study was to find statistically significant data proving that the chest HRCT protocol for evaluation of pediatric patients with BO could include a smaller number of phases, thus reducing the level of radiation exposure. Although CT of the chest has been the subject of a series of recent publications in the Brazilian radiology literature of Brazil^(30-38)^, there have been, to our knowledge, no studies with a similar aim.

Miglioretti et al.^(29)^ suggested that radiation dose-reducing strategies could drastically reduce the incidence of radiation-induced cancer. Our sample included a large number of examinations, which were all performed at the same service, with the same protocols and CT equipment and the same quality, making it more homogeneous and therefore showing more statistically relevant results. However, we excluded some examinations, either because they were performed with a different protocol, were not performed at our institution, were technically poor, or were otherwise unsuitable for radiological analysis, which limited the size of our sample.

It is known that children have an increased lifetime cancer incidence risk, ten times higher than for adults^(39-41)^, not only because of the longer life expectancy but also because they will probably undergo a larger number of CT scans and other examinations involving ionizing radiation during their life. During the period analyzed in the present study, the patients underwent a median of 13.8 examinations. The exposure of the public to radiation from natural sources is 2.4 mSv/year^(42)^, whereas the median effective dose of one chest CT scan in a 5-year-old child is 2.1 mSv^(43)^.

The radiation exposure caused by medical procedures is on the rise and is currently the major artificial source of radiation. In addition, some studies have shown that there have been changes in radiological practices as a result of the creation of new techniques. The use of CT has increased worldwide, from 1–3 procedures/1000 population in the 1977–1980 period to 35 procedures/1000 population in the 1997–2007 period. Although CT accounts for approximately 7% of all radiological procedures world, it accounts for more than 40% of the collective effective dose^(42)^. In the largest population study involving radiation exposure^(44)^, the incidence of all types of cancer was found to be higher for the exposed group than for the unexposed group. At our institution, reducing the radiation dose is a major goal, the health care professionals are continuously informed about the radiation risks, as well as the need for a more conscientious use of radiological procedures, and protocols are constantly being changed in order to achieve that goal. Recent changes in the adult abdominal CT protocols at our institution-modifications in technical aspects of the examinations and in the number of acquisition phases-have reduced the median level of radiation exposure by half^(45)^, benefiting not only the patients, who are exposed to a lower radiation dose, but also the institution, because the scans have become faster and consequently less expensive^(46)^.

Our data show that the inspiratory phase could be excluded from the chest HRCT protocol in children being evaluated for post-ABMT BO. Taking that measure could reduce the radiation exposure in this population by half.
